# Review of Chemistry for Students

**Published:** 1851-07

**Authors:** 


*Review of Chemistry for Students.* Adapted to the courses as taught in the principal medical schools of the United States. By John G. Murphy, M. D. Philadelphia: Lindsay & Blakiston. 1851. 12 mo., pp. 328.

The object and plan of this little work are excellent. It contains some inaccuracies which should be corrected in a second edition. With this exception, it is well calculated to prove a convenient hand book to assist the student in refreshing his memory with the important facts of chemical science. For sale at Butler's.

